# Intra‐ and Interspecific Spatial Temporal Interactions Drive Habitat Selection of Three Sympatric Top Predators

**DOI:** 10.1002/ece3.73753

**Published:** 2026-06-03

**Authors:** Chenbing Chu, Wannian Cheng, Baoxiang Huang, Wentao Zhang, Xinpeng Liu, Shixin Gao, Nathan James Roberts, Heng Bao, Jiayin Gu, Guangshun Jiang

**Affiliations:** ^1^ Feline Research Center of National Forestry and Grassland Administration, College of Wildlife and Protected Area Northeast Forestry University Harbin China; ^2^ Northeast Asia Biodiversity Research Center Northeast Forestry University Harbin China

**Keywords:** habitat selection, intra‐ and interspecific interactions, sympatric coexistence, top predators

## Abstract

The population distribution and habitat selection of top predators are critically important for species conservation and habitat management. While previous studies have identified environmental characteristics and food resources as factors influencing animal habitat selection, the roles of potential intra‐ and interspecific competition have often been overlooked. Utilizing 6 years (2014–2019) of continuous infrared camera trap data from the Hunchun region of China, this study investigated the population density changes of sympatric Amur tigers ( *Panthera tigris altaica* ), Amur leopards ( *P. pardus orientalis* ), and Asiatic black bears ( *Ursus thibetanus* ). Furthermore, Generalized Linear Mixed Models (GLMMs) were employed to characterize the habitat selection patterns of these three species. The results of the study indicated a significant increase in the population density of Amur tigers (*p* = 0.05) and Amur leopards (*p* = 0.035), but no significant change for Asiatic black bears (*p* = 0.86) during this period. Notably, the presence of the three top predators exerted different impacts on the distribution patterns of other species during the population recovery process. Moreover, the results indicate that top predator habitat selection arises from the combined effects of intra‐ and interspecific interactions, environmental characteristics, and prey availability. Specifically, the densities of Amur tigers and leopards influenced their own habitat use, while tiger and leopard densities also affected the habitat selection of Asiatic black bears. Consequently, conservation paradigms should shift from mere prey restoration to the holistic fulfillment of carnivores' fundamental habitat needs and the management of spatial competition, including targeted migration induction efforts for key species to achieve sustainable population growth.

## 1 Introduction

The mechanisms governing the coexistence of species with similar ecological niches in sympatry have long been a central focus of ecological research (Di Bitetti et al. 2010; Ramesh et al. 2012; Li et al. 2024). Ecological niche theory posits that the variety and number of species capable of coexisting within a community are constrained by the similarity of their functional positions within an ecosystem (Jaksic and Marone 2007; Di Bitetti et al. 2010). Beyond niche differentiation, the top‐down effects exerted by carnivore‐dominated top predator communities play a significant role in maintaining ecosystem balance through species interactions and trophic cascades (Hunter and Price 1992; Elmhagen et al. 2010; Colman et al. 2014), and influence community composition and species abundance (Paine 1980, Terborgh et al. 2001; Dorresteijn et al. 2015). However, anthropogenic disturbance and habitat fragmentation intensify interspecies competition (Symes et al. 2018; Kadoya et al. 2022; Carvalho Jr. et al. 2025; Jin et al. 2025), population growth leads to increased species density, which in turn intensifies both intra‐ and interspecific competition, subsequently driving animals to prioritize occupying high‐quality habitats to maximize individual fitness (Stamps and Krishnan 1995; Fedriani et al. 1999; Andheria et al. 2007; Ramesh et al. 2012; Sugimoto et al. 2016).

The Amur tiger ( *Panthera tigris altaica* ), Amur leopard ( *P. pardus orientalis* ), and Asiatic black bear ( *Ursus thibetanus* ) are sympatric top predators distributed across northeast China and the Russian Far East (Robert Steinmetz et al. 2013, Seryodkin et al. 2018). According to the IUCN Red List, these species are classified as Endangered, Critically Endangered, and Vulnerable, respectively (Uphyrkina 2002, Dinerstein et al. 2007, Garshelis and Steinmetz 2020). As keystone carnivores, they maintain the structural and functional stability of ecosystems by suppressing herbivore populations and inhibiting dominant competitors (Ripple et al. 2014; She et al. 2022). Historically, these predators faced severe population declines in China due to habitat loss and a depleted prey base caused by increasing anthropogenic pressures (Yu et al. 2009; Jiang et al. 2015; Wan et al. 2019). Fortunately, recent conservation efforts including the Grain‐for‐Green Program, the National Natural Forest Protection Project, and the establishment of national parks have contributed to the preservation and recovery of wildlife habitats (Jiang et al. 2017; Wang et al. 2023). This population recovery provides a unique context to observe shifts in habitat selection strategies.

Despite this recovery, studies on the relationships between population growth and habitat selection in Amur tigers, Amur leopards, and Asiatic black bears remain scarce. Existing research has predominantly focused on environmental factors and prey availability, often neglecting the influence of intra‐ and interspecific competition intensified by increasing density. Furthermore, such studies typically utilize data collected over short time scales, which limits the ability to observe habitat selection processes during population fluctuations (McLoughlin et al. 2010). To address these gaps, this study utilizes 6 years (2014–2019) of continuous infrared camera trap data from Hunchun, China, to address the population change and habitat selection of these three predators. We test the following two hypotheses: (1) With strengthened conservation efforts, wildlife populations have exhibited an increasing trend, and the presence of apex predators has resulted in distinct distribution patterns of other species across the landscape; and (2) besides environmental and prey factors, intra‐ and interspecific interactions will significantly influence the habitat selection of the three sympatric top predators.

## 2 Methods

### 2.1 Study Area

**FIGURE 1:**
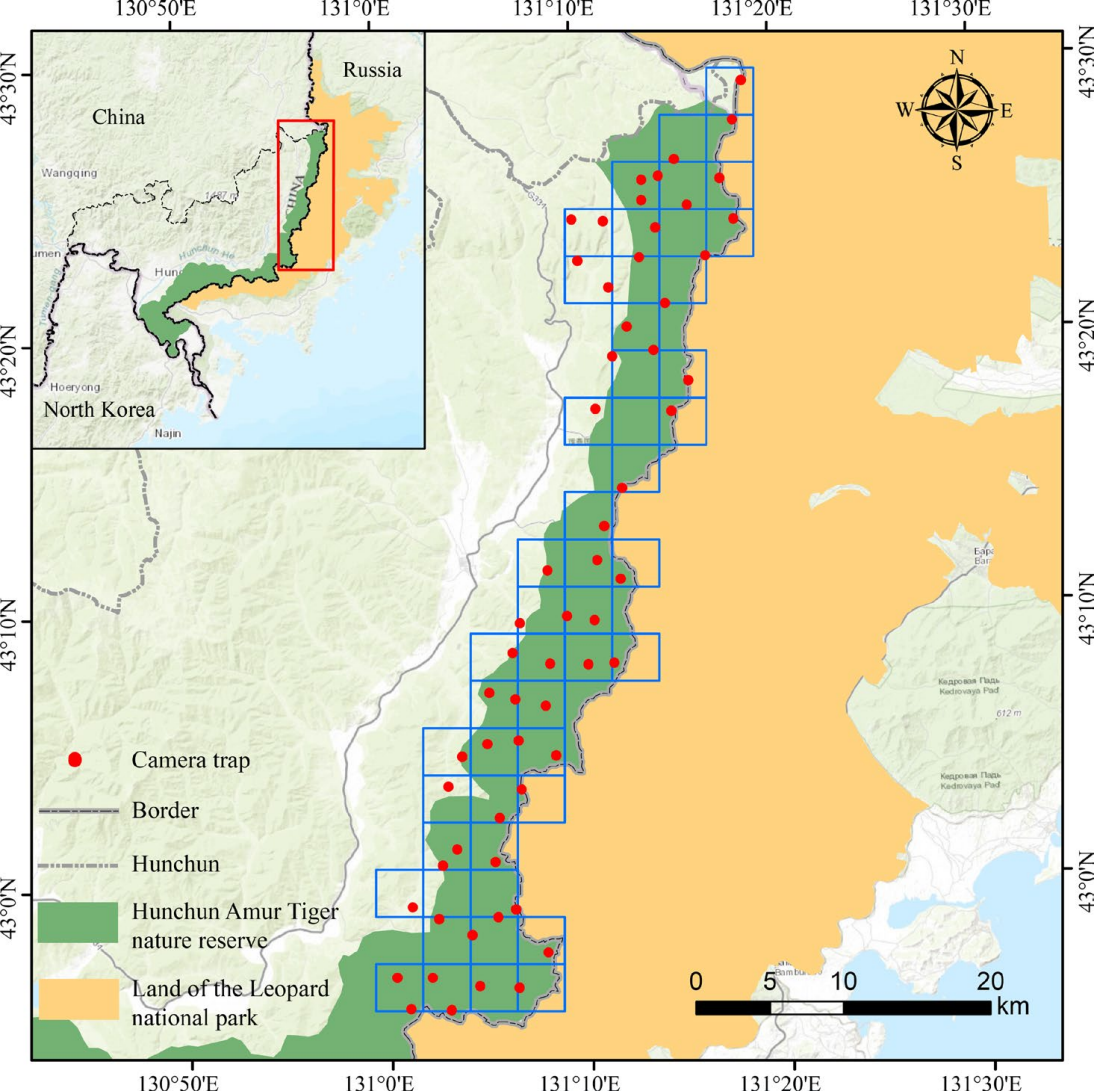
Camera trap distribution map of the study area (*N* = 60). Camera traps were deployed from January 2014 to December 2019.

This study was conducted in Hunchun, Jilin Province, located in northeastern China. The research site was formerly part of the Hunchun National Nature Reserve for Amur Tigers (130°14′08″ ~131°14′44″ E, 42°24′40″ ~43°28′00″ N) (Li et al. 2008). Characterized primarily by low mountainous terrain within the Laoyeling Mountains of the Changbai Mountain range, the study area exhibits elevations ranging from 5 to 1477 m. Its geographical proximity to the Sea of Japan contributes to a mean annual temperature of 5.65°C and an average annual precipitation of 617.9 mm. The regional vegetation cycle is divided into distinct growing (May to October) and nongrowing seasons (November to April) (Li et al. 2008; Liu et al. 2021; Wen et al. 2024). This study area is adjacent to Russia's Land of the Leopard National Park in Primorsky Krai; this area has witnessed increasing cross‐border movements of Amur tiger and leopard populations alongside their demographic recovery (Gu et al. 2018). Serving as a vital habitat for Amur tigers, Amur leopards, and Asiatic black bears (Vitkalova et al. 2018), the region represents one of the most significant international wildlife ecological corridors for future transboundary conservation efforts between China and Russia (Gu et al. 2018) (Figure 1).

The study area boasts abundant wildlife, with key animal species including the Amur tiger, Amur leopard, Asiatic black bear, sika deer ( *Cervus nippon* ), roe deer ( *Capreolus pygargus* ), wild boar ( *Sus scrofa* ), red fox ( *Vulpes vulpes* ), Eurasian badger ( *Meles meles* ), and raccoon dog ( *Nyctereutes procyonoides* ). Dominant forest types consist of oak forests and larch forests, complemented by mixed forests, spruce forests, birch forests, and Korean pine forests. Additionally, the region encompasses scattered villages. As temperatures gradually rise with seasonal changes, local residents engage in activities such as crop cultivation, cattle grazing, and collection of wild vegetables and mushrooms within specific zones of the study area.

### 2.2 Camera Trap Surveys

From 2014 to 2019, camera traps were used to collect wildlife data. In this study, three top predator species and ungulates were the core focal species. We deployed a pair of cameras within each 3.2 × 3.2 km grid; a total of 60 camera trap stations were deployed. This sampling strategy was formulated based on the home range size of ungulates (Ofstad et al. 2016), and was consistent with the long‐term sampling design adopted in this region for many years (Qi et al. 2021; She et al. 2022). This sampling method met the home range requirements of ungulates and other small animals while reducing spatial autocorrelation between cameras (Legendre 1993; Silveira et al. 2003; Qi et al. 2021). During the selection of camera trap stations, we deployed cameras at potential mammal occurrence sites, such as ridges, valleys, trails, forest roads and water‐accessible areas, to maximize the detection probability of wildlife (Wang et al. 2016; She et al. 2022). At each station, one camera was placed on each side of the trail, facing the trail. The camera was fixed on a tree trunk about 40–80 cm above the ground and 3–5 m from the target detection point (Jacobs and Ausband 2018). This not only allowed capturing Amur tigers, Amur leopards, and Asiatic black bears but also enabled photographing ungulates and other small mammals such as foxes, badgers and yellow‐ throated martens and hares. During this period, we performed maintenance on the camera every 3 months, including removing any occlusion in front of the camera, replacing the camera if missing or faulty, copying camera data, and replacing its batteries.

In the processing of infrared camera trap data, the images or videos containing animal and human activities were first screened, identified, classified, and recorded according to the species and human disturbance categories and the time and date that each photo was taken. At the same time, to avoid the repeated recording of data during analysis, photos of individuals of the same species taken more than 0.5 h were recorded as independent valid events (O'Brien et al. 2003).

### 2.3 Population Density Changes and Distribution Patterns of Three Top Predators

Different trophic level mammals show different dispersal abilities, and species with higher trophic levels or stronger dispersal abilities may be captured by multiple camera traps (She et al. 2024). To explain the inherent observation bias in camera trap data and enhance the robustness of data analysis, we used the Random Encounter Model to convert independent photos into more reliable richness estimates (Rowcliffe et al. 2008; She et al. 2024). The calculation formula is as follows:

$$D=\frac{y}{t}\frac{\pi }{v_{i}\;r\left(2+\theta \right)}$$

where *D* is the density of species, *y* is the number of independent photos of species per unit of time, *t* is a unit of time in days, *vi* is the daily movement speed of species *I* (Table S2), *r* is the monitoring radius of the camera, and *θ* is the monitoring angle of the camera.

First, boxplots were used to depict the relative density of six target species (Amur tigers, Amur leopards, Asiatic black bears, roe deer, wild boar, sika deer) in the study area, and the generalized additive model (GAM) was applied to illustrate the interannual changes in their relative density during 2014–2019. Using Amur tigers as an example, camera sites were divided into presence sites and absence sites based on the presence/absence of Amur tigers. After categorizing the camera trap sites based on the presence/absence of Amur tigers, we compared the density changes of other species (excluding Amur tigers) at the two types of sites during 2014–2019. Identical methodology was applied to Amur leopards and Asiatic black bears.

### 2.4 Modeling the Habitat Selection of Three Sympatric Top Predators

We downloaded the 30 m digital elevation DEM from the geospatial data cloud (https://www.gscloud.cn); the vegetation type, villages, farmlands, rivers, and roads data came from the National Geographic Information Resource Catalog Service System (http://www.webmap.cn), and used ArcGIS 10.8.2 to extract slope and aspect data from DEM, and then used the Multivalue Extraction to Points tool to extract elevation, slope, aspect values. The distance from the camera monitoring point to villages, farmlands, rivers, roads, and specific forest types was extracted using the Nearest Neighbor Analysis tool for subsequent analysis.

Firstly, to ensure the uniformity of dimensions and value ranges among different variables, the variables were standardized (Jiang et al. 2010). To reduce the correlation between independent variables, the Pearson correlation test was performed for altitude, slope, aspect, and distance to rivers, farmland, villages, roads, and various forest types using the “corSelect” function in R. To improve the accuracy of the model, when the correlation coefficient is greater than 0.7, it is considered that there is a strong correlation between the variables, and the remaining variables of collinearity are removed for model construction (Ramsay et al. 2003; Dormann et al. 2013; Li et al. 2017). The Generalized Linear Mixed Model (GLMM) was used to construct an optimal model affecting the habitat selection of Amur tigers, Amur leopards, and Asiatic black bears. Taking the Amur tiger habitat selection model as an example, the presence or absence of Amur tigers was used as the response variable, the relative density of Amur tigers in year Nt−1 , the density of Amur leopards, Asiatic black bears, roe deer, wild boars, sika deer, human disturbance, and various variables in year Nt were used as explanatory variables to quantify the overall effects of competitive pressure, prey factors, and environmental factors, and the year was used as a random effect to quantify the variation of Amur tigers' habitat selection under different interannual differences.

At the same time, the R package “YawMMF” was used to fit the GLMM, the “dredge” function in the “MuMIn” package was used to perform full subset regression on the model, and the model selection was carried out according to the Akaike Information Criterion adjusted for small sample size (AICc). Model with △AICc < 2 were retained, the Akaike weight (ωi) of each model was calculated, and the model with the lowest AICc was selected (Guthery et al. 2003; Brewer et al. 2016). r.squaredGLMM() was used to calculate the marginal *R* ^2^ (*R* ^2^ *m*) and conditional *R* ^2^ (*R* ^2^ *c*) of the optimal model (Zhang et al. 2020; Lai 2023), and the “confint()” function was used to calculate the confidence intervals of each factor in the model. The construction of the habitat selection model for the Amur leopard and Asiatic black bear was the same as that of the Amur tiger, and the above data analysis was performed in R software (R Core Team 2024).

## 3 Results

### 3.1 Population Density Changes and Distribution Patterns of Three Top Predators

From January 2014 to December 2019, 120,085 camera monitoring days were sampled within the study area (Table A1). During this period, the Amur tiger was recorded 453 times, the Amur leopard 341 times, and the Asiatic black bear 94 times.

**FIGURE 2:**
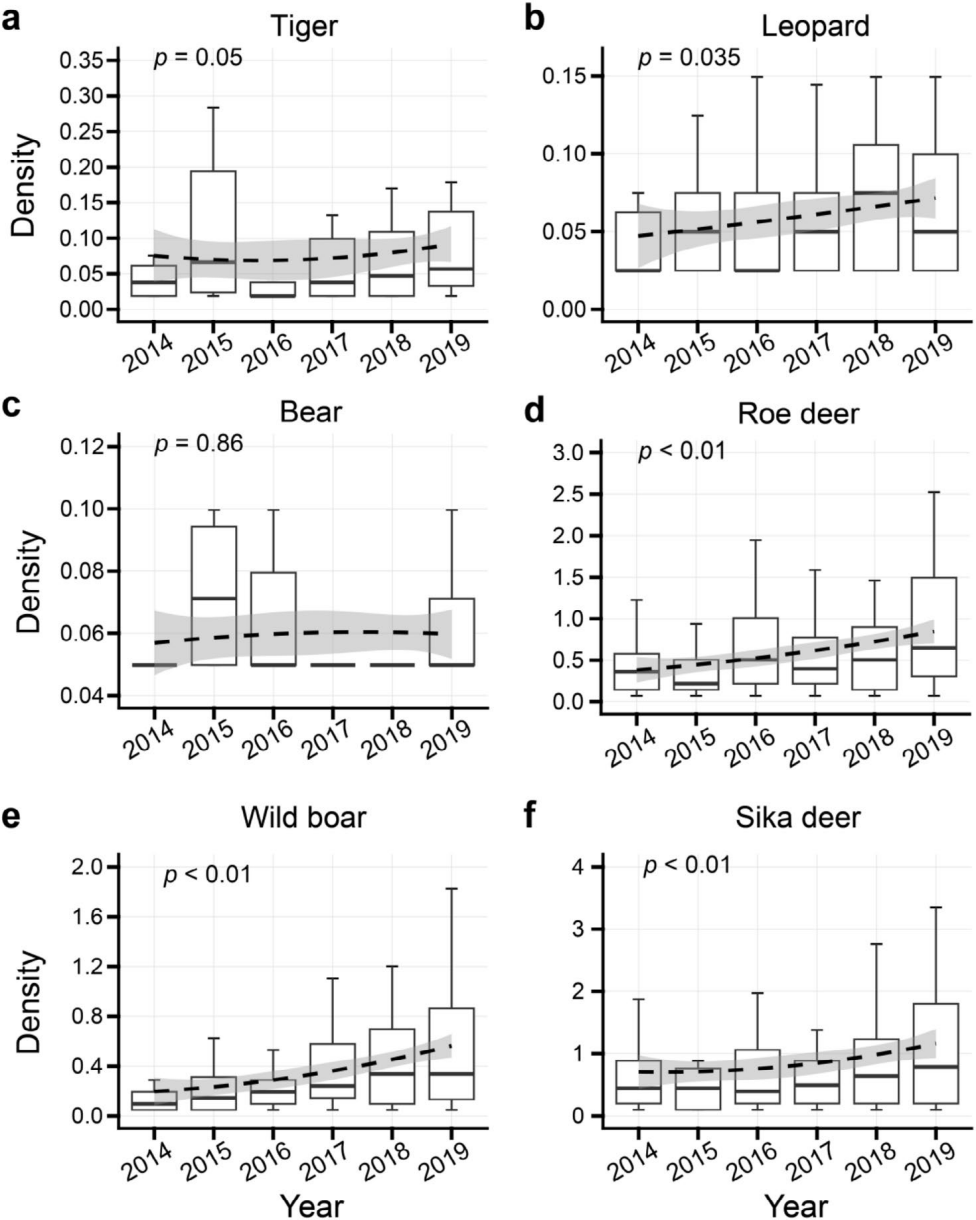
Temporal trends in population density of Amur tigers, Amur leopards, Asiatic black bears, and sympatric species from 2014 to 2019, with the *x‐*axis representing the year and the *y*‐axis representing the relative average density values of each species. Boxplots show the distribution of density estimates for each year. The dashed line shows the trend in species density from 2014 to 2019, fitted using a GAM, with the shaded area indicates the 95% confidence interval.

By comparing the relative average density changes of Amur tigers, Amur leopards, Asiatic black bears, and ungulates from 2014 to 2019, the results showed that the relative density of Amur leopards (*p* = 0.035), roe deer (*p* < 0.01), wild boars (*p* < 0.01), and sika deer (*p* < 0.01) had significant growth trends; Amur tigers (*p* = 0.05) also had growth trends from 2016 to 2019, while the relative density of Asiatic black bears did not change significantly (*p* = 0.86; Figure 2).

According to the occurrence data of Amur tigers, Amur leopards, and Asiatic black bears, the camera trap stations were divided into presence points and absence points to analyze the density change trends of the three top predators.

**FIGURE 3:**
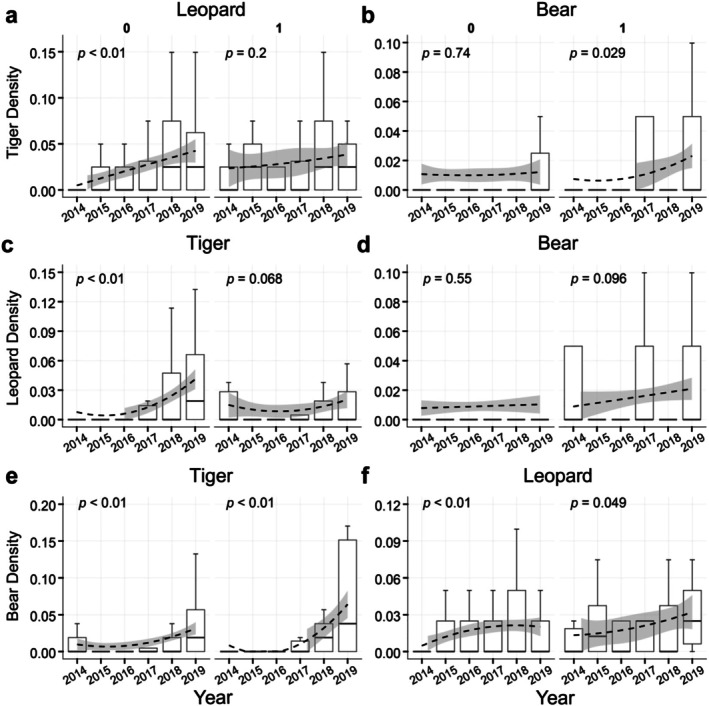
The population density trends of two other top predators were observed in areas where Amur tiger, Amur leopard, and Asiatic black bear were absent and present stations from 2014 to 2019. The *x*‐axis represents the year, and the *y*‐axis represents the relative average density values of each species. Boxplots show the distribution of density estimates for each year. The dashed line shows the trend in species density from 2014 to 2019, fitted using a GAM, with the shaded area indicates the 95% confidence interval. The “0” represents absent camera trap stations, “1” represents present camera trap stations.

The results demonstrated a significant increase in the relative density of Amur leopards (*p* < 0.01) at the camera trap stations where Amur tigers are absent, while the relative density change trend of Asiatic black bears (*p* = 0.45) was not obvious. At the presence camera trap stations of Amur tigers, the relative density of Asiatic black bears (*p* = 0.029) increased significantly, while the relative density of Amur leopards (*p* = 0.2) did not change significantly (Figure 3a,b). For the ungulate prey and human activity, the results indicated a significant relationship between the relative density of roe deer (*p*_0_ < 0.01, *p*_1_ < 0.01) and wild boar (*p*_0_ < 0.01, *p*_1_ = 0.032), while the relative density trend of sika deer at Amur tigers' absence sites increased significantly (*p* = 0.01), but not obvious at presence sites (*p* = 0.91). No significant difference in human activities was detected between the two camera site types (*p*_0_ = 0.20, *p*_1_ = 0.96) (Figure S1).

At the camera trap stations where Amur leopards absence, the relative density of the Amur tiger (*p* = 0.01) increased significantly, while the relative density of the Asiatic black bear (*p* = 0.55) did not change significantly. At the presence camera trap stations of the Amur leopard, the relative density changes trend of the Amur tiger (*p* = 0.068) and the Asiatic black bear (*p* = 0.096) were not obvious (Figure 3c,d). For the ungulate prey and human activity, the results indicated a significant relationship between the relative density of roe deer (*p*_0_ < 0.01, *p*_1_ < 0.01) and wild boar (*p*_0_ < 0.01, *p*_1_ < 0.01), while the relative density trend of sika deer at Amur leopards' absence sites increased significantly (*p* = 0.022), but not obvious at presence sites (*p* = 0.086). No significant difference in human activities was detected between the two camera site types (*p*_0_ = 0.90, *p*_1_ = 0.62) (Figure S2).

The relative densities of Amur tigers (*p =* 0.01) and Amur leopards (*p*_0_ = 0.01, *p*_1_ = 0.049) increased significantly in the presence and absence of camera trap stations of Asiatic black bears (Figure 3e,f). For the ungulate prey and human activity, the results indicated a significant relationship between the relative density of roe deer (*p*_0_ < 0.01, *p*_1_ < 0.01), sika deer (*p*_0_ = 0.027, *p*_1_ = 0.021), and wild boar (*p*_0_ < 0.01, *p*_1_ = 0.019). No significant difference in human activities was detected between the two camera site types (*p*_0_ = 0.44, *p*_1_ = 0.69) (Figure S3).

### 3.2 Habitat Selection of Three Sympatric Top Predators Under Intra‐ and Interspecific Interactions

Amur tigers include 5 models, Amur leopards include 2 models, and Asiatic black bears include 4 models (Table S3).

The results of the optimal GLMM model for Amur tiger habitat selection showed that Amur tigers showed negative selection for elevation (*p* = 0.015), and Amur tigers tended to appear in lower altitude areas. Amur tigers also showed negative selection for wild boar density (*p* = 0.056). Meanwhile, the presence of Amur tigers exhibited positive selection for *t*−1 Amur tiger density (*p* = 0.015), roe deer density (*p* = 0.012), and human activities (*p* = 0.006), as well as negative selection for distance to farmland (*p* = 0.01). However, the presence or absence of Amur tigers showed random selection for sika deer density (*p* = 0.155, 95% CI = −0.093 ~ 0.841). In the Amur tiger model, *R* ^2^ *m* was 0.423, *R* ^2^ *c* was 0.525, and the *R* ^2^ value attributed to year as a random effect was 0.102, indicating that the habitat selection of Amur tigers did not vary substantially across years.

The results of the GLMM model showed that the presence of Amur leopards was significantly positively correlated with *t*−1 Amur leopard density (*p* = 0.012), bear density (*p* = 0.034), distance to rivers (*p* = 0.003), and larches (*p* = 0.001), and significantly negatively correlated with elevation (*p* = 0.004) and distance to Korean pines (*p* = 0.012). However, roe deer density was subject to random selection (*p* = 0.059, 95% CI = −0.015 ~ 0.567). In the Amur leopard model, *R* ^2^ *m* and *R* ^2^ *c* were both 0.176, and year, as a random effect, did not contribute to the variation in the response variable, indicating that the habitat selection of Amur leopards did not vary substantially across years.

The results of the optimal GLMM model showed that the presence of Asiatic black bears exhibited positive selection for Amur tiger density (*p* ≤ 0.001), Amur leopard density (*p* = 0.009), and distance to oak forests (*p* ≤ 0.001), as well as negative selection for distance to national highways (*p* = 0.002). Meanwhile, Asiatic black bears showed random selection for aspect (*p* = 0.082, 95% CI = −0.036 ~ 0.700) and distance to birch forests (*p* = 0.106, 95% CI = −0.818 ~ 0.062). In the Asiatic black bear model, *R* ^2^ *m* was 0.157 and *R* ^2^ *c* was 0.160, with the *R* ^2^ value attributed to year as a random effect being 0.003 (Table 1), indicating that the habitat selection of Asiatic black bears did not vary substantially across years.

**TABLE 1:** Optimal habitat selection model for Amur tiger, Amur leopard, and Asiatic black bear, where *R* ^2^ *m* represents marginal *R* ^2^, *R* ^2^ *c* represents conditional *R* ^2^, and the difference between *R* ^2^ *c* and *R* ^2^ *m* is the *R* ^2^ contributed by years as random effects. *t*−1 denotes the density of the species in year *t*−1.

Species	Covariates	Estimate	*Pr* (*>|z|*)	95% CI	*R* ^2^
Tiger	(Intercept)	−1.028	0.036*	−2.270 ~ 0.121	*R* ^2^ *m* = 0.423 *R* ^2^ *c* = 0.525
	Elevation	−0.700	0.015*	−1.287 ~ −0.149	
	*t*−1 Tiger density	0.860	0.015*	0.269 ~ 1.666	
	Sika deer density	0.336	0.155	−0.093 ~ 0.841	
	Distance to farmland	0.690	0.01**	0.181 ~ 1.232	
	Roe deer density	0.623	0.012*	0.159 ~ 1.128	
	Human activity	0.490	0.006**	0.143 ~ 0.851	
	Wild boar density	−0.918	0.056^¶^	−1.927 ~ 0.053	
Leopard	(Intercept)	−0.132	0.339	−0.402 ~ 0.139	*R* ^2^ *m* = 0.176 *R* ^2^ *c* = 0.176
	*t*−1 Leopard density	0.444	0.012*	0.120 ~ 0.814	
	Elevation	−0.426	0.004**	−0.721 ~ −0.142	
	Distance to river	0.451	0.003**	0.157 ~ 0.757	
	Distance to korean pine	−0.456	0.012*	−0.820 ~ −0.109	
	Distance to larch	0.593	0.001**	0.240 ~ 0.964	
	Roe deer density	0.274	0.059^¶^	−0.015 ~ 0.567	
	Black bear density	0.307	0.034*	0.032 ~ 0.613	
Black bear	(Intercept)	−1.679	≤ 0.001***	−2.221 ~ −1.237	*R* ^2^ *m* = 0.157 *R* ^2^ *c* = 0.160
	Tiger density	0.592	≤ 0.001***	0.264 ~ 0.973	
	Leopard density	0.415	0.009**	0.100 ~ 0.730	
	Aspect	0.325	0.082¶	−0.036 ~ 0.700	
	Distance to national highways	−0.602	0.002**	−0.999 ~ −0.234	
	Distance to oak	0.808	≤ 0.001***	0.433 ~ 1.223	
	Distance to birch	−0.361	0.106	−0.818 ~ 0.062	

*Note:* “***”, “**”, “*”, and “¶” indicate *p* < 0.001, *p* < 0.01, *p* < 0.05, and *p* < 0.1, respectively.

## 4 Discussion

### 4.1 Population Density Changes and Distribution Patterns of Three Top Predators

In this study, the results confirm that aside from the slow growth of the Asiatic black bear population, the populations of Amur leopards, roe deer, wild boar, and sika deer exhibited a significant increasing trend, Amur tigers also exhibited a increasing trend (Figure 2). These findings are in agreement with earlier research on the population trends of Amur tigers and leopards (Jiang et al. 2017; Qi et al. 2021; Wang et al. 2023). Additionally, our research reveals that the presence or absence of Amur tigers, Amur leopards, and Asiatic black bears results in varying spatial distribution patterns among other species. While all three are apex predators, their ecological regulatory roles are nonidentical, within the sympatric ecosystem, Amur tigers dominated interspecific competition, whereas Amur leopards and Asiatic black bears acted as subordinate competitors (Harihar et al. 2011; Hojnowski et al. 2012), resulting in a lack of significant population density increase for Amur leopards in areas with Amur tigers, whereas Amur leopards exerted minimal impact on Amur tigers. Dietary analyses indicate that Amur tigers primarily prefer wild boar and sika deer, while Amur leopards preferentially consume sika deer and roe deer (Gu et al. 2018; Yang et al. 2018). However, in areas where Amur leopards were present, the population density of sika deer increased at a slower rate, whereas the growth of roe deer and wild boar remained unaffected. Concurrently, the results indicated an increase in Asiatic black bear population density at sites where Amur tigers and leopards were present. This is likely attributable to Asiatic black bears scavenging at tiger and leopard kill sites to access food resources (Seryodkin et al. 2018). Although Asiatic black bears are also top predators, their role in ecological regulation appears to be weaker than that of Amur tigers and Amur leopards.

### 4.2 Habitat Selection of Three Sympatric Top Predators Under Intra‐ and Interspecific Interactions

The abundance and spatial distribution of prey populations significantly influence the habitat selection of top predators (Silveira et al. 2003), while habitat quality, resource availability, and intra‐ and interspecific interactions are also contributing factors (Fretwell and Lucas 1969; Rosenzweig 1981; Karanth et al. 2004; Santora et al. 2011). As territorial species, Amur tigers, Amur leopards, and Asiatic black bears maintain relatively fixed home ranges, highlighting the importance of considering conspecific density in the habitat selection of territorial animals (O'Neil et al. 2019). This is particularly relevant during population fluctuations, as shifting densities can alter how species respond to various environmental factors in habitat selection models, thereby influencing model outcomes (O'Neil et al. 2019). Our results demonstrate that the population density of Amur tigers and leopards significantly influenced their habitat selection. This likely occurs because increased intraspecific pressure results in a greater number of individuals lacking established home ranges. In response, resident territory owners may intensify territorial behaviors, reinforcing their use of specific habitats to defend their territories. Therefore, understanding the spatial competition pressures imposed by growing populations, along with the associated territorial behaviors employed to secure habitats, is crucial for the conservation of these territorial species.

Previous research indicates that under elevated population density and competitive pressure, territorial species increase their utilization of secondary habitats (Morris 1994). For instance, O'Neil applying Resource Selection Probability Functions (RSPF) demonstrated that increasing wolf population density led to the prioritization of high‐quality habitat by established packs, subsequently displacing other individuals into lower‐quality habitats. Consistent with this, our GLMM analysis revealed that the habitat selection of Amur tigers and Amur leopards was significantly influenced by density constraints, whereas that of Asiatic black bears was not. This study was conducted in a China‐Russia border region characterized by numerous transboundary movements of Amur tigers and Amur leopards. For these territorial felids, the positive association between their conspecific density and habitat selection may be driven by competition between resident individuals and floaters. In contrast, the Asiatic black bear population has exhibited no significant density change or notable growth in recent years; consequently, its habitat selection was more strongly influenced by the presence of Amur tigers and Amur leopards. Although omnivorous, Asiatic black bears acquire food resources by scavenging remains from Amur tiger and leopard kills. This scavenging behavior often occurs with a temporal lag following the predation event (Seryodkin et al. 2018).

Previous dietary studies have identified roe deer, wild boar, and sika deer as primary prey for Amur tigers and leopards, with tigers exhibiting a strong preference for wild boar, followed by sika deer and roe deer, and leopards preferring roe deer while avoiding wild boar (Yang et al. 2018). However, our model results revealed a significant positive correlation only between Amur tiger occurrence and roe deer density. When year was included as a random factor, a significant positive correlation was identified between Amur tiger occurrence and roe deer density, whereas associations with sika deer and wild boar density were nonsignificant. Additionally, a significant positive correlation was observed between Amur tiger occurrence and both anthropogenic disturbance and proximity to farmland. This suggests that increasing tiger density could exacerbate human‐tiger conflict, underscoring the need for managers to implement spatiotemporal segregation strategies to mitigate such conflicts (Cheng et al. 2024). A significant correlation was also detected between Amur leopard and Asiatic black bear in habitat selection. This may stem from the substantial spatial niche differentiation between Amur leopards and tigers, coupled with the Asiatic black bear's broad activity range, which results in greater spatial niche overlap between leopards and bears. This study demonstrates that intraspecific competition significantly influences the habitat selection of Amur tigers and leopards, while the presence of tigers and leopards also exerts a significant influence on Asiatic black bears habitat use.

## 5 Conclusion

A comprehensive analysis of 6 year infrared camera trap data from the Hunchun region reveals a significant recovery in the populations of Amur tigers and Amur leopards, whereas the Asiatic black bear population remained relatively stable during the same period. Despite this recovery, the observed deceleration in growth rates for all three species suggests heightened intraspecific competition within the limited habitat. This study further demonstrates that beyond traditional environmental factors and prey availability, the population recovery and spatial distribution of top predators are critically influenced by intra‐ and interspecific competition. Specifically, the density of tigers and leopards exerts a significant influence on their own habitat selection, while the habitat selection of Asiatic black bears is distinctly shaped by the density of these two superior felids. These findings provide novel insights that can inform future conservation strategies for protecting sympatric large carnivores in Northeast China. It is imperative to evaluate population change in conjunction with intra‐ and interspecific competitive pressures, especially considering the diverse environmental requirements of species within their respective habitats. Consequently, the conservation paradigm should shift from mere prey restoration to the holistic fulfillment of carnivores' fundamental habitat resource needs. To address the challenges posed by competitive pressures, targeted migration induction efforts should be implemented for key species to achieve sustainable population growth for the Amur tiger, Amur leopard, and Asiatic black bear.

## Author Contributions

**Chenbing Chu:** conceptualization (lead), data curation (lead), formal analysis (lead), methodology (lead), visualization (lead), writing – original draft (lead). **Wannian Cheng:** conceptualization (equal), data curation (equal), investigation (equal), software (equal). **Baoxiang Huang:** data curation (equal), methodology (equal), visualization (equal). **Wentao Zhang:** data curation (equal), software (equal), visualization (equal). **Xinpeng Liu:** investigation (equal), software (equal). **Shixin Gao:** investigation (equal), software (equal). **Nathan James Roberts:** conceptualization (equal), investigation (equal), methodology (equal), writing – original draft (supporting). **Heng Bao:** conceptualization (supporting), methodology (supporting), supervision (equal), writing – review and editing (supporting). **Jiayin Gu:** conceptualization (equal), methodology (equal), supervision (equal). **Guangshun Jiang:** conceptualization (lead), funding acquisition (equal), writing – review and editing (equal).

## Funding

This research was funded by the National Key Research and Development Program of China (2023YFF1305000).

## Conflicts of Interest

The authors declare no conflicts of interest.

## Supporting information


**Table S1:** Camera trap stations data for the study area from 2014 to 2019, each station deployed a pair of cameras; a total of 60 camera trap stations were deployed. An aberrant camera point suggests that the camera at a given monitoring location is malfunctioning or is missing, resulting in missing camera point data.
**Table S2:** Daily movement distances of each species.
**Table S3:** GLMM models for Habitat Selection of Amur tigers, Amur leopards, and Asiatic black bears. The model with △AICc < 2 was selected for presentation (△AICc represents the difference from the top model). For the species of tiger, leopard, bear, roe deer, wild boar, sika deer, and human activity, the values represent species density in the current year. The value “*t*−1” denotes density in the previous year, referred to as “year *t*−1.” The distances for various tree species indicate the distance to the forest dominated by that tree species.
**Figure S1:** Temporal trends in population density of Roe deer, Sika deer, Wild boar and human activity for the nonoccurrence and occurrence stations of Amur tiger from 2014 to 2019, with the *x*‐axis representing the year and the *y*‐axis representing the relative average density values of each species. Boxplots show the distribution of density estimates for each year. The dashed line shows the trend in species density from 2014 to 2019, fitted using a GAM, with the shaded area indicates the 95% confidence interval. The “0” represents absent camera trap stations, “1” represents persent camera trap stations.
**Figure S2:** Temporal trends in population density of Roe deer, Sika deer, Wild boar and human activity for the nonoccurrence and occurrence stations of Amur leopard from 2014 to 2019, with the *x*‐axis representing the year and the *y*‐axis representing the relative average density values of each species. Boxplots show the distribution of density estimates for each year. The dashed line shows the trend in species density from 2014 to 2019, fitted using a GAM, with the shaded area indicates the 95% confidence interval. The “0” represents absent camera trap stations, “1” represents persent camera trap stations.
**Figure S3:** Temporal trends in population density of Roe deer, Sika deer, Wild boar and human activity for the nonoccurrence and occurrence stations of Asiatic black bear from 2014 to 2019, with the *x*‐axis representing the year and the *y*‐axis representing the relative average density values of each species. Boxplots show the distribution of density estimates for each year. The dashed line shows the trend in species density from 2014 to 2019, fitted using a GAM, with the shaded area indicates the 95% confidence interval. The “0” represents absent camera trap stations, “1” represents persent camera trap stations.

## Data Availability

All the required data are uploaded as Supporting Information.
