# Supplementary material for: Intra‐ and Interspecific Spatial Temporal Interactions Drive Habitat Selection of Three Sympatric Top Predators
Source: Ecol Evol. 2026 Jun 3;16(6):e73753. doi: 10.1002/ece3.73753 (PMC13240163; doi:10.1002/ece3.73753)

**APPENDIX**

**APPENDIX TABLE 1**. Camera trap stations data for the study area from 2014 to 2019, each station deployed a pair of cameras; a total of 60 camera trap stations were deployed. An aberrant camera point suggests that the camera at a given monitoring location is malfunctioning or is missing, resulting in missing camera point data.

| Year | Number of normally operating Camera trap stations | Number of abnormally operating Camera trap stations | Total number of Camera trap stations |
| --- | --- | --- | --- |
| 2014 | 53 | 7 | 60 |
| 2015 | 53 | 7 | 60 |
| 2016 | 56 | 4 | 60 |
| 2017 | 56 | 4 | 60 |
| 2018 | 52 | 8 | 60 |
| 2019 | 59 | 1 | 60 |

**APPENDIX TABLE 2.** Daily movement distances of each species

| Species | Daily travel distance (km) | References |
| --- | --- | --- |
| Tiger | 14.48 | (Rozhnov et al., 2011) |
| Leopard | 11.00 | (Hubel et al., 2018) |
| Bear | 5.50 | (Bartoń et al., 2019) |
| Roe deer | 3.80 | (Danilkin, 1995) |
| Sika deer | 2.78 | (Stephens et al., 2006) |
| Wild boar | 5.70 | (Cavazza et al., 2023) |

Rozhnov V V, Hernandez-Blanco J A, Lukarevskiy V S, et al. Application of satellite collars to the study of home range and activity of the Amur tiger (*Panthera tigris altaica*)[J]. Biology Bulletin, 2011, 38(8): 834-847.

Hubel T Y, Golabek K A, Rafiq K, et al. Movement patterns and athletic performance of leopards in the Okavango Delta[J]. Proceedings of the Royal Society B: Biological Sciences, 2018, 285(1877): 20172622.

Bartoń K A, Zwijacz-Kozica T, Zięba F, et al. Bears without borders: Long-distance movement in human-dominated landscapes[J]. Global Ecology and Conservation, 2019, 17: e00541.

Danilkin A A. Capreolus pygargus[J]. Mammalian Species, 1995(512): 1-7.

Stephens P A, Zaumyslova O Yu, Miquelle D G, et al. Estimating population density from indirect sign: track counts and the Formozov–Malyshev–Pereleshin formula[J]. Animal Conservation, 2006, 9(3): 339-348.

Cavazza S, Brogi R, Apollonio M. Sex-specific seasonal variations of wild boar distance traveled and home range size[J]. Current Zoology, 2023: zoad021.

**APPENDIX TABLE 3**. GLMM models for Habitat Selection of Amur tigers, Amur leopards, and Asiatic black bears. The model with △AICc < 2 was selected for presentation (△AICc represents the difference from the top model). For the species of tiger, leopard, bear, roe deer, wild boar, sika deer, and human activity, the values represent species density in the current year. The value "t-1" denotes density in the previous year, referred to as "year t-1." The distances for various tree species indicate the distance to the forest dominated by that tree species.

| Species | Model description | df | logLik | AICc | △AICc | ωi |
| --- | --- | --- | --- | --- | --- | --- |
| Tiger | t-1 Tiger + Elevation + Sika deer + Roe deer + Wild boar + Farmland + Human activity + (1\|year) | 9 | -108.278 | 235.296 | 0.000 | 0.298 |
|  | t-1 Tiger + Elevation + Roe deer + Wild boar + Farmland + Human activity + (1\|year) | 8 | -109.415 | 235.420 | 0.124 | 0.280 |
|  | t-1 Tiger + Elevation + Sika deer + Roe deer + Wild boar+ Farmland +Bear+ Human activity +(1\|year) | 10 | -107.794 | 236.496 | 1.200 | 0.164 |
|  | t-1 Tiger +Elevation+ Roe deer + Wild boar + Farmland + Human activity + Bear +(1\|year) | 9 | -108.988 | 236.717 | 1.421 | 0.146 |
|  | t-1 Tiger + Elevation + Leopard + Sika deer + Roe deer + Wild boar + Farmland + Human activity +(1\|year) | 10 | -108.176 | 237.261 | 1.966 | 0.112 |
| Leopard | t-1 Leopard + Elevation + Bear + Roe deer +River + Korea pink + Larch+ (1\|year) | 9 | -153.270 | 325.280 | 0.000 | 0.331 |
|  | t-1 Leopard + Elevation + Bear + River+ Korea pink + Larch + (1\|year) | 8 | -154.366 | 325.323 | 0.043 | 0.324 |
| Black bear | Tiger + Leopard + Oak + Slope+ Highway + Birch +(1\|year) | 8 | -104.658 | 225.907 | 0.000 | 0.342 |
|  | Tiger + Leopard + Oak + Slope + Highway +(1\|year) | 7 | -106.047 | 226.551 | 0.644 | 0.248 |
|  | Tiger + Leopard + Oak + Highway + Birch +(1\|year) | 7 | -106.209 | 226.875 | 0.968 | 0.211 |
|  | Tiger + Leopard + Oak + Highway + (1\|year) | 6 | -107.325 | 226.993 | 1.086 | 0.199 |

Note: AICc: Small-sample corrected AIC; △AICc: Difference from the top model; ωi: Model weight.

**APPENDIX FIGURE 1.** Temporal trends in population density of Roe deer, Sika deer, Wild boar and human activity for the non-occurrence and occurrence stations of Amur tiger from 2014 to 2019, with the x-axis representing the year and the y-axis representing the relative average density values of each species. Boxplots show the distribution of density estimates for each year. The dashed line shows the trend in species density from 2014 to 2019, fitted using a GAM, with the shaded area indicates the 95% confidence interval. The ‘0’ represents non-occurrence camera trap stations, ‘1’ represents occurrence camera trap stations.


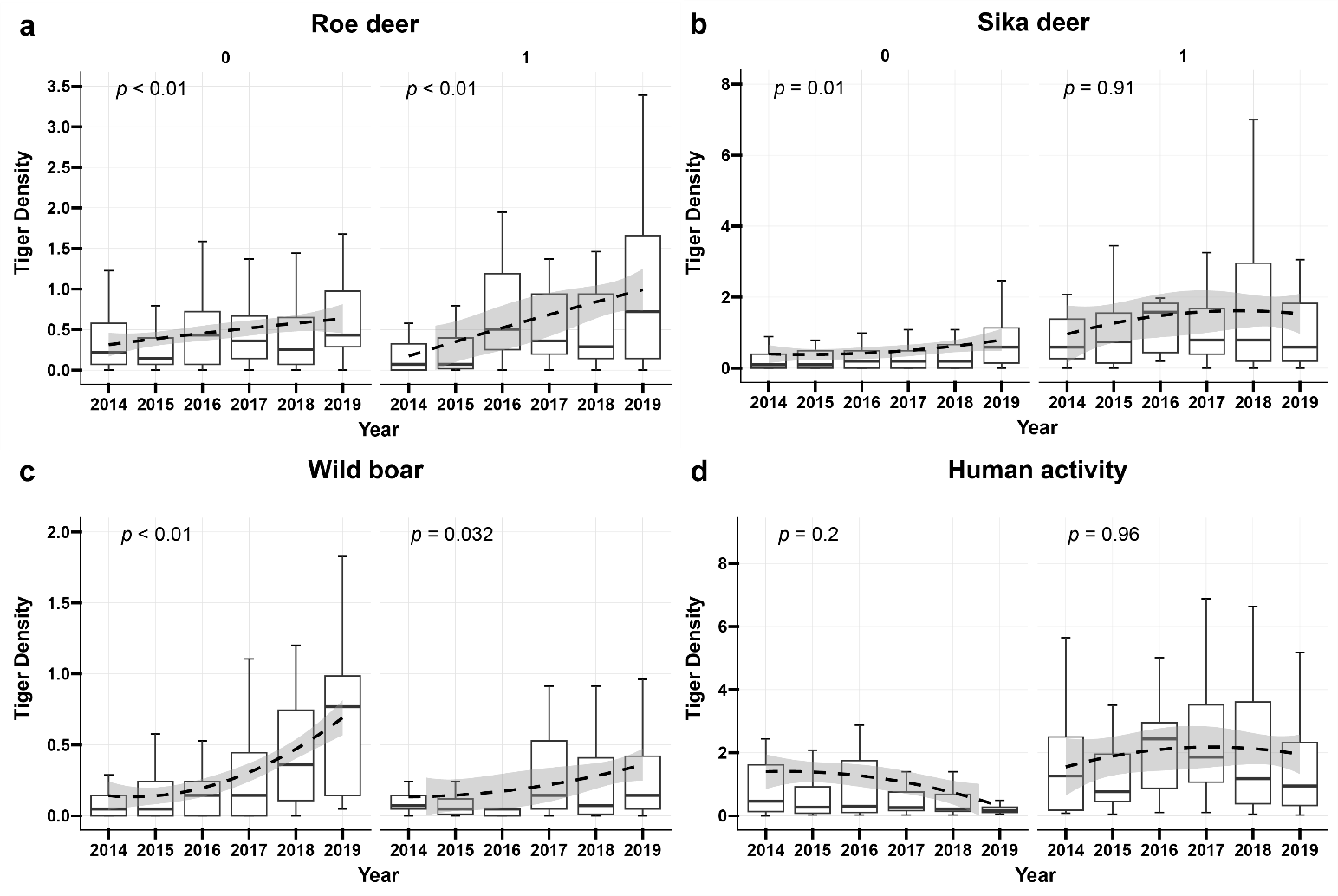


**APPENDIX FIGURE 2**. Temporal trends in population density of Roe deer, Sika deer, Wild boar and human activity for the non-occurrence and occurrence stations of Amur leopard from 2014 to 2019, with the x-axis representing the year and the y-axis representing the relative average density values of each species. Boxplots show the distribution of density estimates for each year. The dashed line shows the trend in species density from 2014 to 2019, fitted using a GAM, with the shaded area indicates the 95% confidence interval. The ‘0’ represents non-occurrence camera trap stations, ‘1’ represents occurrence camera trap stations.


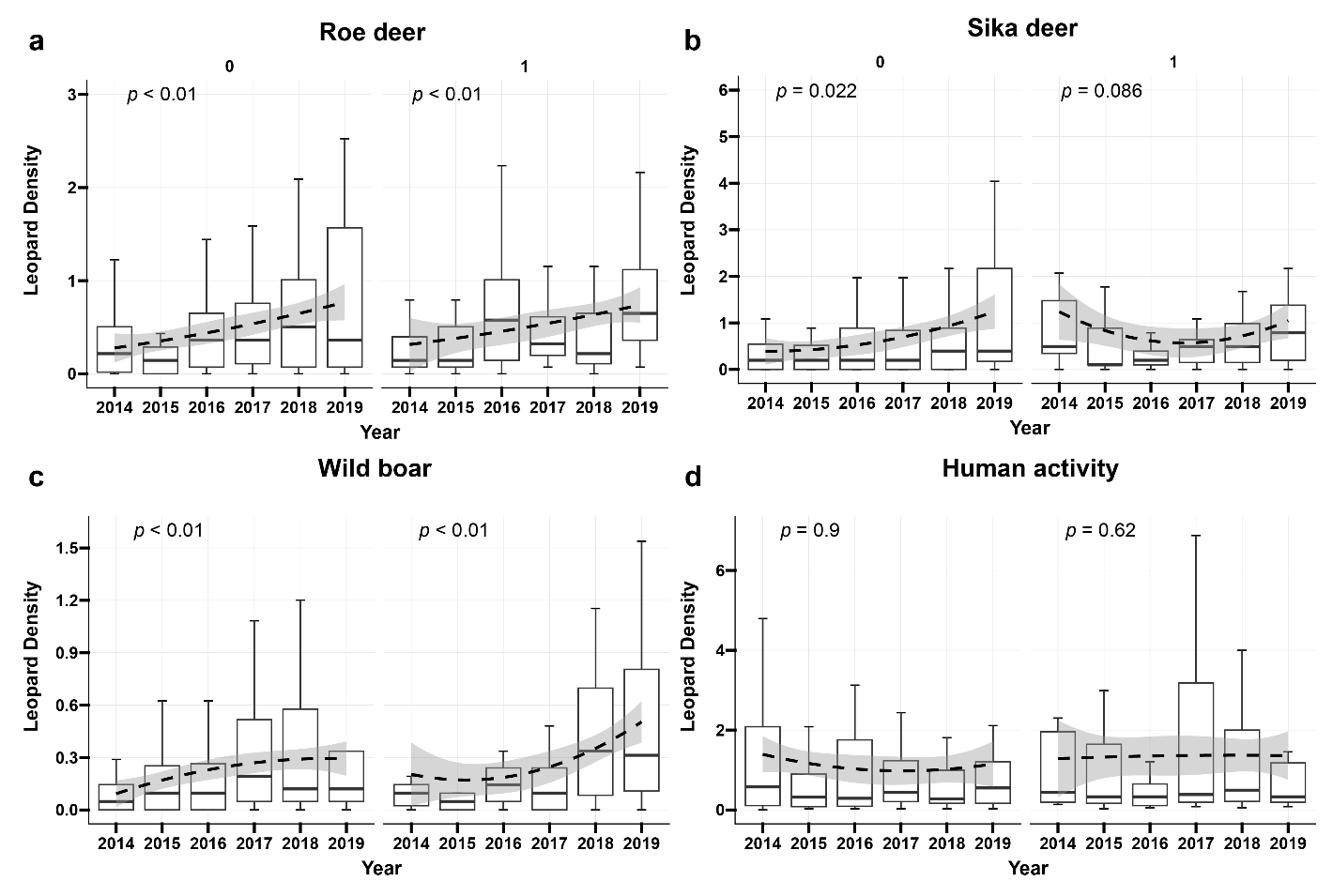


**APPENDIX FIGURE 3**. Temporal trends in population density of Roe deer, Sika deer, Wild boar and human activity for the non-occurrence and occurrence stations of Asiatic black bear from 2014 to 2019, with the x-axis representing the year and the y-axis representing the relative average density values of each species. Boxplots show the distribution of density estimates for each year. The dashed line shows the trend in species density from 2014 to 2019, fitted using a GAM, with the shaded area indicates the 95% confidence interval. The ‘0’ represents non-occurrence camera trap stations, ‘1’ represents occurrence camera trap stations.


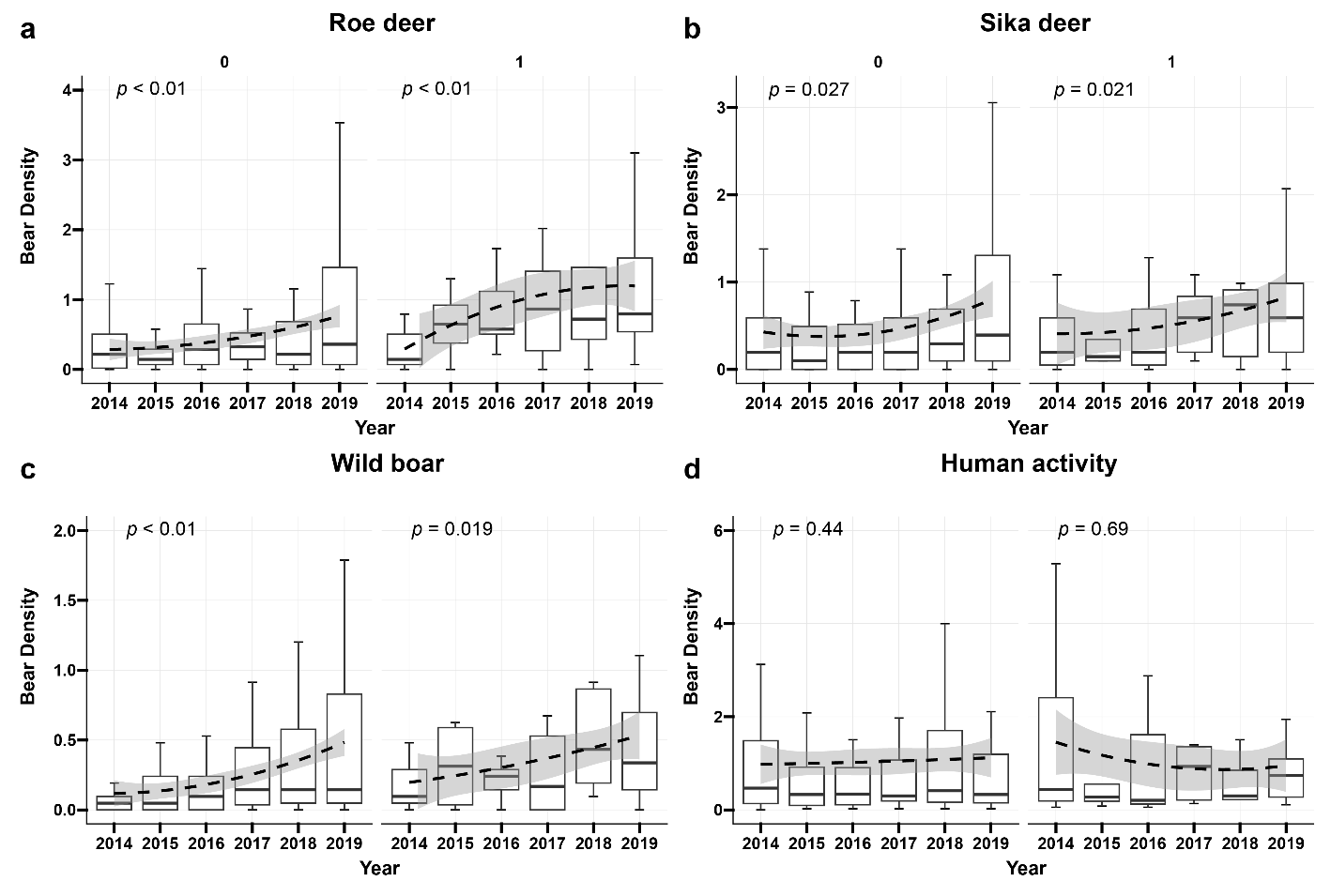

Supplement: Supplementary file 1 — Table S1: Camera trap stations data for the study area from 2014 to 2019, each station deployed a pair of cameras; a total of 60 camera trap stations were deployed. An aberrant camera point suggests that the camera at a given monitoring location is malfunctioning or is missing, resulting in missing camera point data. Table S2: Daily movement distances of each species. Table S3: GLMM models for Habitat Selection of Amur tigers, Amur leopards, and Asiatic black bears. The model with △AICc < 2 was selected for presentation (△AICc represents the difference from the top model). For the species of tiger, leopard, bear, roe deer, wild boar, sika deer, and human activity, the values represent species density in the current year. The value “t−1” denotes density in the previous year, referred to as “year t−1.” The distances for various tree species indicate the distance to the forest dominated by that tree species. Figure S1: Temporal trends in population density of Roe deer, Sika deer, Wild boar and human activity for the nonoccurrence and occurrence stations of Amur tiger from 2014 to 2019, with the x‐axis representing the year and the y‐axis representing the relative average density values of each species. Boxplots show the distribution of density estimates for each year. The dashed line shows the trend in species density from 2014 to 2019, fitted using a GAM, with the shaded area indicates the 95% confidence interval. The “0” represents absent camera trap stations, “1” represents persent camera trap stations. Figure S2: Temporal trends in population density of Roe deer, Sika deer, Wild boar and human activity for the nonoccurrence and occurrence stations of Amur leopard from 2014 to 2019, with the x‐axis representing the year and the y‐axis representing the relative average density values of each species. Boxplots show the distribution of density estimates for each year. The dashed line shows the trend in species density from 2014 to 2019, fitted using a GAM, with the sha [file ECE3-16-e73753-s001.docx]
